# Virtual Non-Contrast versus True Native in Photon-Counting CT: Stability of Density of Upper Abdominal Organs and Vessels

**DOI:** 10.3390/diagnostics14111130

**Published:** 2024-05-29

**Authors:** Florian Haag, Shanice S. Emmrich, Alexander Hertel, Johann S. Rink, Dominik Nörenberg, Stefan O. Schoenberg, Matthias F. Froelich

**Affiliations:** Department of Radiology and Nuclear Medicine, University Medical Center Mannheim, Heidelberg University, Theodor-Kutzer-Ufer 1–3, 68167 Mannheim, Germany

**Keywords:** photon-counting computed tomography, virtual non-contrast, multiphasic computed tomography, protocol optimization

## Abstract

The clinical use of photon-counting CT (PCCT) allows for the generation of virtual non-contrast (VNC) series from contrast-enhanced images. In routine clinical practice, specific issues such as ruling out acute bleeding require non-contrast images. The aim of this study is to evaluate the use of PCCT-derived VNC reconstructions in abdominal imaging. PCCT scans of 17 patients including early arterial, portal venous and native sequences were enrolled. VNC reconstructions have been calculated. In every sequence and VNC reconstruction, 10 ROIs were measured (portal vein, descending aorta, inferior vena cava, liver parenchyma, spleen parenchyma, erector spinae muscle, subcutaneous adipose tissue, first lumbar vertebral body, air, and psoas muscle) and density values were compared. The VNC reconstructions show significant changes in density compared to the contrast-enhanced images. However, there were no significant differences present between the true non-contrast (TNC) and any VNC reconstructions in the observed organs and vessels. Significant differences (*p* < 0.05) between the measured mean density values in the TNC versus VNC reconstructions were found in fat and bone tissue. The PCCT-derived VNC reconstructions seemed to be comparable to the TNC images, despite some deviations shown in the adipose tissue and bone structures. However, the further benefits in terms of specific clinical issues need to be evaluated.

## 1. Introduction

Computed tomography (CT) is an essential imaging modality in routine clinical practice, which is constantly being optimized by technical improvements. One of the latest and most revolutionary innovations is the use of photon-counting detectors, which enable the direct measurement of photons and their energy. In contrast, traditional CT detectors rely on scintillator technology and include a separate step involving the generation of visual light, which is measured in a photodiode [1]. Through the direct measurement of photons, PCCT has improved the spatial resolution and the ability to obtain energy information from the measurement of photon energy. This novel detector technology delivers additional information, which results in improved dose efficiency and spectral information [1,2]. As energy information from the measurement of photon energy is detected, PCCT enables spectral reconstructions of the acquired data sets [3]. Using these, Virtual Monoenergetic Images (VMIs) can be generated with post-processing techniques [4]. In scans acquired using contrast media, spectral information can be used to create a virtual non-contrast (VNC) series. Subsequently, iodine-containing contrast media is identified and subtracted from the image series [5].

In clinical routine, there is a demand for non-contrast, i.e., native imaging in several specific clinical questions such as bleeding exclusion or evaluation of vessel grafts [6]. If the VNC sequences can substitute true non-contrast (TNC) sequences, the scan could be shortened and radiation exposure for the patient can be reduced.

Previous studies using Dual Energy CT (DECT)-derived VNC reconstructions have shown promising results. A high level of consistency between VNC and TNC was shown in many cases, such as, for example, in the imaging of brain structures [7], abdominal organs [8,9,10,11] and vessels [12]. Similarly, studies on DECT-derived data sets have repeatedly described the direct clinical benefits of VNC. For example, intramural aortic haematomas can be diagnosed using VNC reconstructions [13]. Also, VNC reconstructions from DECT and PCCT have been shown to be useful for coronary calc screening in cardiac CT [14,15]. However, DECT-derived VNC reconstructions also seem the be imitated in coronary calc screening, as the calc volumes measured in VNC tend to be smaller, like those measured in TNC (DOI: 10.3348/kjr.2016.17.3.321). Additionally, several studies have shown that DECT-derived VNC reconstructions cannot totally substitute TNC images. In the imaging of the neck, a significant variation in the density of the thyroid tissue has been demonstrated between VNC reconstructions and TNC [16]. In the detection of kidney stones, the accuracy of DECT-derived VNC reconstructions was also limited by the surrounding iodine-enhanced contrast [17]. Nevertheless, DECT-derived VNC reconstructions seem to be good additions to conventional image data even if they should also be evaluated critically.

Looking at PCCT, there is a need for studies evaluating the potential of VNC reconstructions. Further studies by Schoenbeck et al. [18] and Niehoff et al. [19] have already described the potential of VNC to substitute TNC in abdominal imaging using PCCT. However, PCCT is a novel technology and the number of patients which can be included in comparative studies is limited. In this context, there is still a need for additional studies dealing with the comparability of VNC reconstructions and TNC images in studies using standardised scan parameters. Also, the usability of VNC sequences for specific clinical issues in PCCT has to be further evaluated.

The aim of the presented study is to further evaluate and validate the application of VNC reconstructions in abdominal imaging, which is a promising approach in abdominal PCCT imaging. Additionally, this work will investigate if there is any difference between VNC reconstructions derived from the arterial or venous contrast phases.

## 2. Materials and Methods

### 2.1. Patient Cohort

This retrospective single-centre study was approved by the local institutional review board and was conducted in accordance with the Helsinki declaration [20]. The following inclusion criteria were applied: (I.) age ≥18 years; (II.) multiphasic CT of the upper abdomen (native, early arterial, and portal venous contrast phase); (III.) PCCT performed; (IV.) patients gave written and informed consent. Exclusion criteria were (I.) age <18 years; (II.) study incomplete or aborted; (III.) previous administration of contrast media on the same day. According to inclusion and exclusion criteria, a total of 17 patients were retrospectively enrolled in this study from December 2021 to July 2023.

### 2.2. CT Scan and Contrast Protocol

A clinical, CE-marked photon-counting CT scanner (NAEOTOM ALPHA, Siemens Healthineers AG, Forchheim, Germany) was used for all examinations. All scans were performed at 120 kVP tube voltage with adaptive tube current (CARE keV IQ). Rotation time was 0.25 s and pitch was set to 1.5. After native imaging of the abdomen, 60 mL iodine-based contrast agent (Iomeprol 350 mg/mL, Bracco Imaging Deutschland GmbH, Konstanz, Germany) was injected at a rate of 4.0 mL/second followed by 30 mL of saline chaser. Image acquisition was started using bolus tracking in the descending aorta at a threshold of 100 HU. After acquisition of early arterial contrast phase images, additional series were acquired in the portal venous contrast phase (60 s delay).

**Figure 1:**
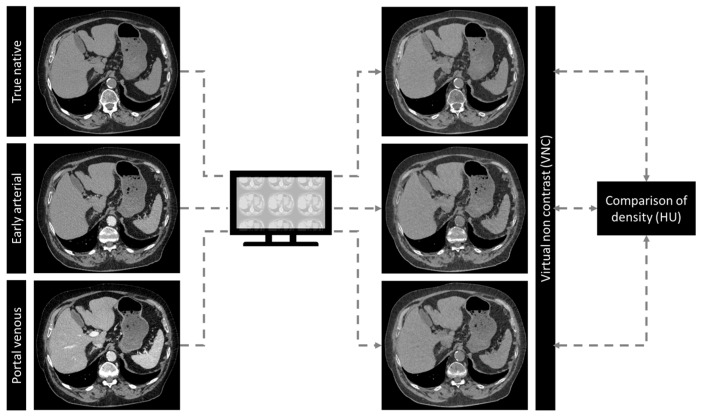
Principles of virtual non-contrast (VNC) reconstructions. Patients with triphasic photon-counting CT (PCCT) scans of the abdomen (true non-contrast, early arterial and portal venous) have been enrolled. VNC reconstructions have been performed from true non-contrast, early arterial and portal venous contrast phase and compared to true non-contrast.

Reconstruction was performed with a dedicated algorithm and a fixed kernel using medium denoising strength (Quantitative Iterative Reconstruction QR40 and Q3). All images were reconstructed on a 512 × 512 matrix with a slice thickness and section increment of 1.0 mm and 0.5 mm, respectively. Additionally, corresponding VNC reconstructions of native (VNCn), early arterial (VNCa) and portal venous (VNCv) contrast phase were created in the vendor console (Syngo.Via, version VB60A, Siemens Healthineers AG, Forchheim, Germany). Figure 1 illustrates post-processing of the acquired images.

### 2.3. Structured Measurements

**Figure 2:**
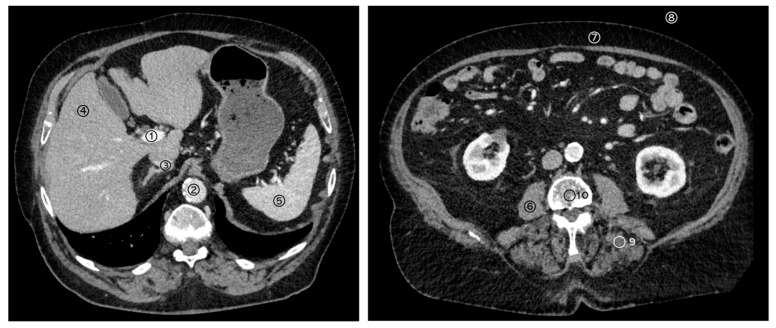
Overview of the measured structures. Circular regions of interest (ROI) were placed in the portal vein (1), in the descending aorta (2), in the inferior vena cava (3), in the liver parenchyma (4), in the spleen parenchyma (5), in the psoas muscle (6), in the subcutaneous adipose tissue (7), in the surrounding air (8), in the erector spinae muscle (9) and in the first lumbar vertebral body (10).

A standardized and structured measurement workflow was performed on polyenergetic (T3D reconstructions) images of the abdomen in native, early arterial and portal venous contrast phase and also in the corresponding VNC reconstructions (VNCn, VNCa and VNCv). According to the standardized measurement workflow, circular 2D regions of interest (ROI) with 1cm diameter were placed in the portal vein (PV), in the descending aorta (DA), in the inferior vena cava (IVC), in the liver parenchyma, in the spleen parenchyma, in the erector spinae muscle (ESM), in the subcutaneous adipose tissue (SAT), in the first lumbar vertebral body (L1), in the surrounding air ventral of the abdomen and in the psoas muscle (PM). In case the target structure was smaller than 1 cm, the size of the circular ROI was adjusted by the reader accordingly. A detailed overview of the measured structures is given in Figure 2. All measurements were performed by the same observer.

### 2.4. Statistical Analysis

Statistical analysis was performed with R statistics (R Statistics, Version 4.1.0, R Core Team, Vienna, Austria)) [21]. Mean values and standard deviation (SD) were calculated for quantitative variables; percentages (%) were calculated for categorical variables. Comparison tables were created with the tableone package (version 0.13.2). Boxplots were created with ggplot2 (version 3.3.5). Bland–Altman plots were created with the BlandAltmanLeh package (version 0.3.1). An additional comparison of the mean values was performed by Kruskal–Wallis test using SPSS (Statistical Package for Social Science, version 29.0.0.0, IBM, Armonk, NY, USA).

## 3. Results

### 3.1. Patient Cohort

The presented retrospective single-centre study included 17 patients (6 female), in accordance with the mentioned inclusion criteria. The mean age of the patients included was 75.65 years. The examinations were performed to examine several clinical questions (staging of oncological disease: 9, vessel evaluation: 7 and bleeding examination: (1). An overview of the patient cohort is provided in Table 1.

### 3.2. Structured Measurement

**Figure 3:**
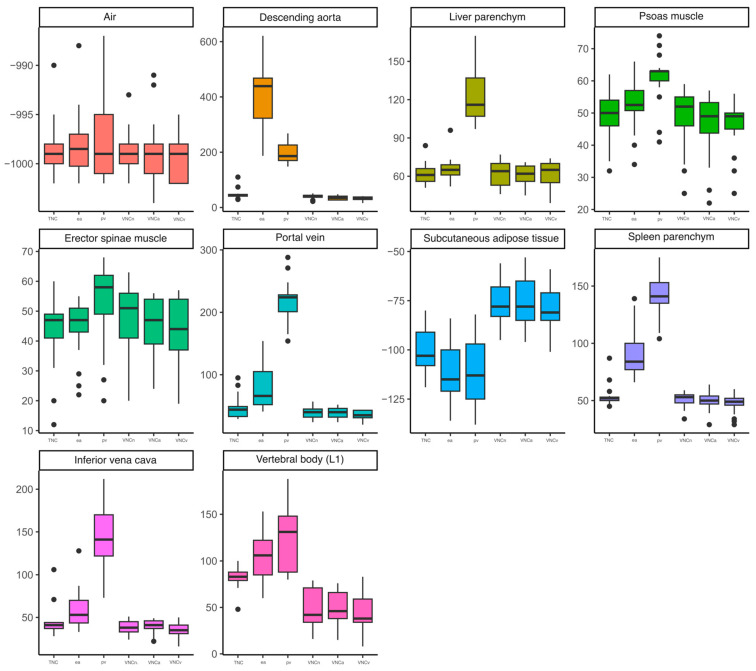
Mean HU values of the measured ROI, illustrated as boxplots. Abbreviations: native-derived virtual non-contrast (VNCn), early arterial (ea), ea-derived VNC (VNCa), portal venous (pv) and pv-derived VNC (VNCv).

**Figure 4:**
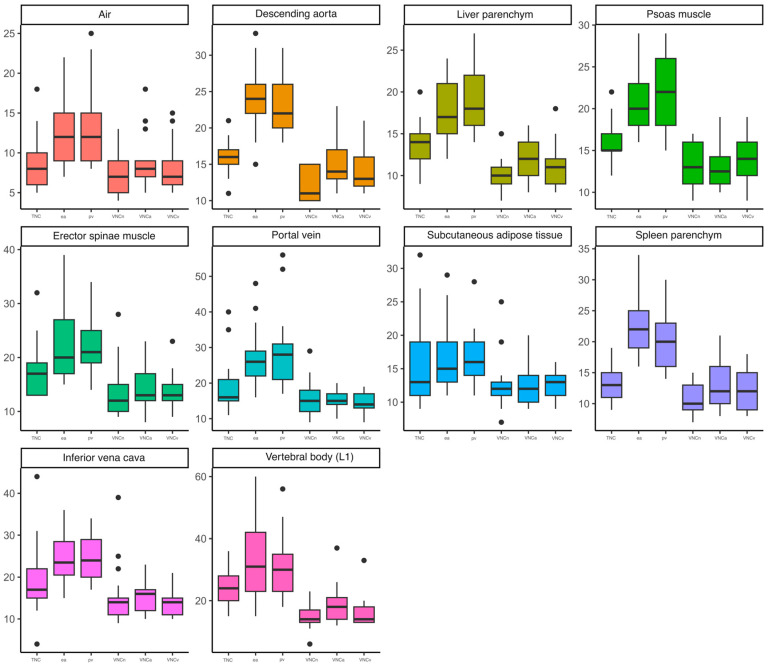
Standard deviation of measured ROI, illustrated as boxplots. Abbreviations: native-derived virtual non-contrast (VNCn), early arterial (ea), ea-derived VNC (VNCa), portal venous (pv) and pv-derived VNC (VNCv).

**Figure 5:**
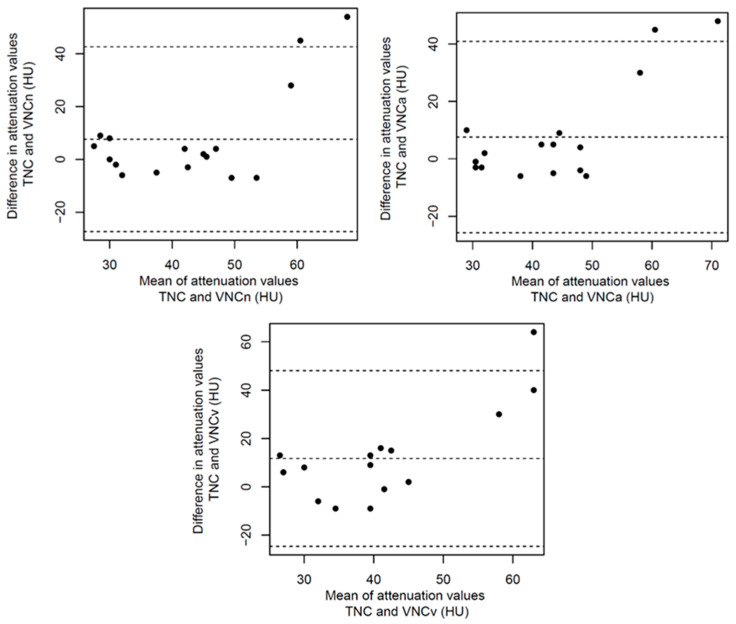
Bland–Altman plots of difference in attenuation values in the portal vein. True non-contrast (TNC) is plotted against virtual non-contrast reconstructions (VNC) derived from TNC (VNCn), early arterial contrast phase (VNCa) and portal venous contrast phase (VNCv). The differences in attenuation values are plotted against mean attenuation values of both measurements (TNC vs. VNC).

**Figure A1:**
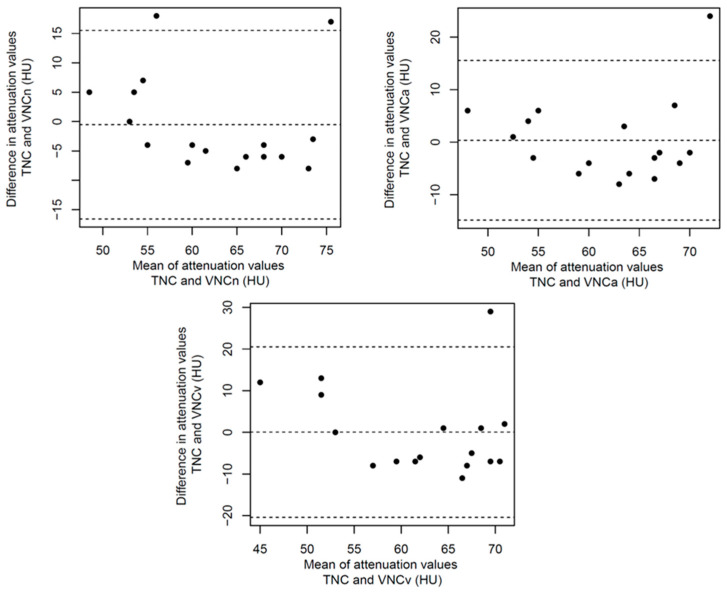
Bland–Altman plots of difference in attenuation values in the liver. True non-contrast (TNC) is plotted against virtual non-contrast reconstructions (VNC) derived from TNC (VNCn), early arterial contrast phase (VNCa) and portal venous contrast phase (VNCv). The differences in attenuation values are plotted against mean attenuation values of both measurements (TNC vs. VNC).

**Figure A2:**
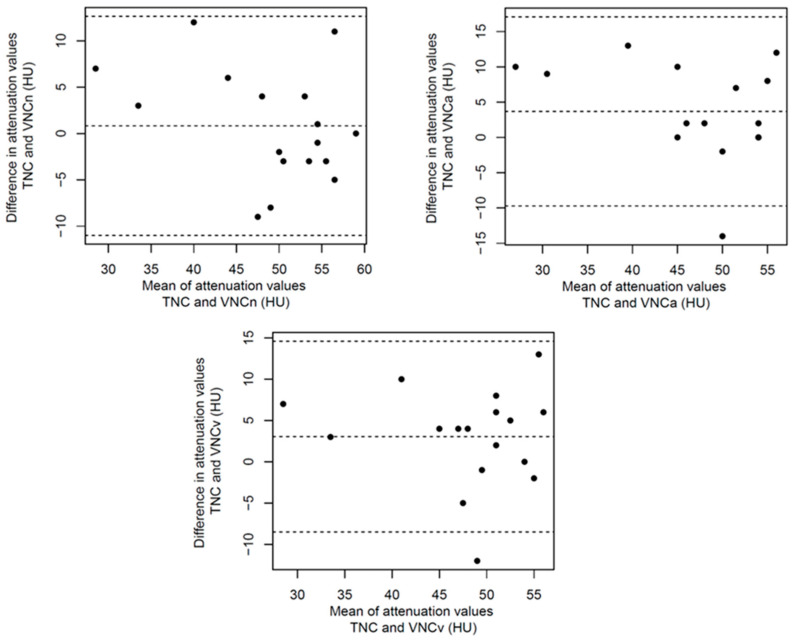
Bland–Altman plots of difference in attenuation values in the psoas muscle. True non-contrast (TNC) is plotted against virtual non-contrast reconstructions (VNC) derived from TNC (VNCn), early arterial contrast phase (VNCa) and portal venous contrast phase (VNCv). The differences in attenuation values are plotted against mean attenuation values of both measurements (TNC vs. VNC).

**Figure A3:**
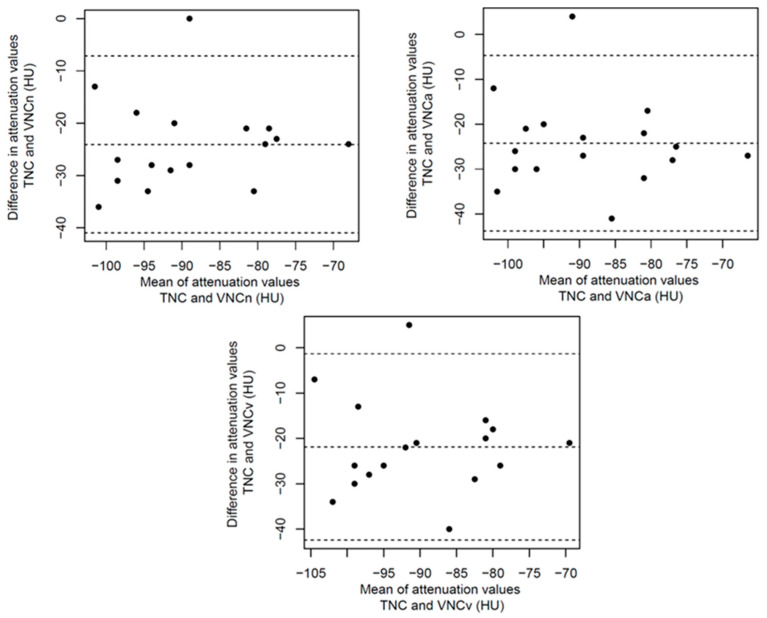
Bland–Altman plots of difference in attenuation values in the subcutaneous adipose tissue. True non-contrast (TNC) is plotted against virtual non-contrast reconstructions (VNC) derived from TNC (VNCn), early arterial contrast phase (VNCa) and portal venous contrast phase (VNCv). The differences in attenuation values are plotted against mean attenuation values of both measurements (TNC vs. VNC).

**Figure A4:**
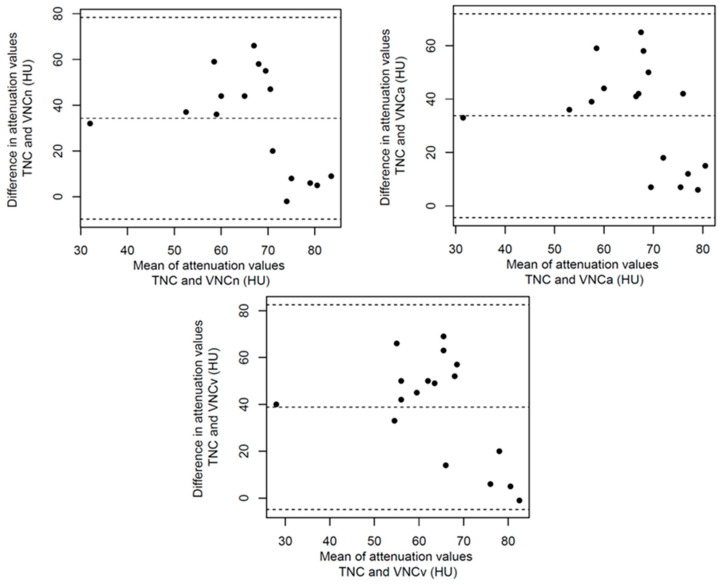
Bland–Altman plots of difference in attenuation values in the first lumbar vertebral body. True non-contrast (TNC) is plotted against virtual non-contrast reconstructions (VNC) derived from TNC (VNCn), early arterial contrast phase (VNCa) and portal venous contrast phase (VNCv). The differences in attenuation values are plotted against mean attenuation values of both measurements (TNC vs. VNC).

Based on the used measurement scheme, the mean values and SD of CT attenuation in HU were measured in all the patients using polyenergetic images (true non-contrast, early arterial and portal venous) and in the corresponding VNC reconstructions (VNCn, VNCa and VNCv), resulting in a total of 1020 ROI. The measured mean values and standard deviations are given in Table 2. Compared to the mean values measured using the polyenergetic TNC images, these were significantly higher than the mean values measured using the polyenergetic portal venous contrast phase in the PV, VCI, liver parenchyma, spleen parenchyma, ESM and PM. In the corresponding VNC reconstructions, there were no significant differences observed compared to the polyenergetic TNC. The mean values of the DA and spleen parenchyma measured in the polyenergetic early arterial contrast phase were significantly higher than the mean values measured in the polyenergetic TNC. In the VNCa reconstructions, the measured mean values of the DA and spleen parenchyma showed no significant differences compared to the TNC reconstructions. The mean values of the SAT and L1 were significantly lower in all the observed VNC reconstructions (VNCn, VNCa and VNCv) compared to the polyenergetic TNC. Accordingly, there were no significant differences observed between the TNC and contrast-enhanced image series (early arterial and portal venous). The mean values of the measured density in air did not show any significant differences between the TNC images, the other observed contrast phases nor the VNC images. An overview of the calculated significance levels is given in Table 3. The boxplots given in Figure 3 illustrate that the mean value measurements of the early arterial and portal venous contrast phase differ especially in the vessels (DA, PV, VCI), muscles (PM, ESM) and parenchymal organs (liver and spleen) from the TNC reconstructions. In these structures, the VNC reconstructions of the different contrast phases also graphically approximate the TNC reconstructions. When looking at the SAT and L1, the different contrast phases differ less from each other. However, there is a considerable difference between the original sequences and the calculated VNC sequences. There were no significant differences between the VNCa and VNCv images in any of the observed structures (all *p* > 0.05). The mean values of the measured SD are given in Table 4 and illustrated in Figure 4. The boxplots in Figure 4 also show differences from the TNC to the early arterial and portal venous contrast phase reconstructions. Nevertheless, the differences between the measurements are also particularly noticeable when looking at the structures that absorb contrast media (DA, PV, VCI, PM, ESM, liver and spleen). Looking at the SAT and L1, the boxplots also illustrate the significant difference between the TNC and the VNC sequences. For better visualisation, the mean values were illustrated as Bland–Altman plots (Figure 5, Figure A1, Figure A2, Figure A3 and Figure A4), whereby the difference between the attenuation of the TNC and VNC (VNCn, VNCa and VNCv) reconstructions was plotted on the *y*-axis and the mean of the attenuation values was plotted on the *x*-axis. As an example, for the abdominal organs, the mean values of the density measurements of the liver are illustrated in Figure A1. This shows that the VNCn, VNCa and VNCv images are comparable to the TNC images regarding the liver attenuation. The mean values of the vessels are exemplified in Figure 5 by a Bland–Altman plot of the measurements of the portal vein. Figure 5 shows that the mean values measured in the portal vein are comparable between the TNC and VNC images. Considering the psoas muscle, the Bland–Altman plot of the measured mean values also indicate that the VNCn, VNCa and VNCv images are comparable to the TNC images (Figure A2). The plots of the SAT (Figure A3) and L1 (Figure A4) show, in contrast to the other plots, a wide scatter, which suggests that the VNC images are not comparable to the TNC images in these cases. In comparison, the illustrations of the Bland–Altman plots emphasise the results described and illustrated above.

## 4. Discussion

The presented study demonstrates the potential of PCCT-derived VNC reconstructions to substitute TNC sequences and add clinical value to abdominal imaging.

In routine clinical practice, there are several use cases for TNC sequences, e.g., bleeding evaluation, detection of urinary stones or exclusion of intraluminal haematoma [22,23,24]. In some cases, however, these specific questions arise during further clinical procedures, when a CT with contrast medium has already been performed without a non-contrast phase. In these specific cases, it could be helpful to use the spectral reconstructions of PCCT and calculate a VNC sequence. However, there is a need for comparative studies between VNC and TNC to evaluate if they are comparable in these specific clinical issues.

In DECT, the potential of VNC in comparison to TNC is well examined, indicating that VNC can substitute TNC in several specific cases. However, in DECT, the use of VNC to detect fatty liver tissue [25,26] to differentiate adrenal adenomas from adrenal metastases [27], to detect urolithiasis [28,29] and to discriminate intraperitoneal haematoma [30] has been described previously. Despite these promising findings, pitfalls of VNC in DECT have been described. Lethi et al. were able to show a higher attenuation of vessels and higher image noise levels in VNC reconstructions derived from arterial sequences compared to TNC [31]. Also, other authors have described differences in density values measured in vessels between VNC and TNC [12,32]. Furthermore, Durieux et al. illustrated that VNC is not capable of replacing TNC images in abdominal imaging. Their work showed substantial differences in the measured attenuation for fluid, fat and renal tissues [33]. In summary, VNC demonstrated potential in DECT for substituting TNC in specific and selected cases. Nevertheless, these reconstructions have limitations and must be used carefully. There are only a few studies that describe the use of VNC in PCCT. Due to its higher spatial resolution and image quality, the VNC images derived from PCCT may have an improved quality and the potential to improve CT diagnostics in routine clinical practice [1]. In line with the findings in DECT, further work was able to demonstrate the good clinical applicability of VNC images in the evaluation of liver and spleen tissue [18], well in line with our findings. Other preparatory work has described differences between TNC and VNC reconstructions in terms of the measured attenuation values [19]. This work also showed differences between the TNC and VNC images, but these were only significant in L1 and SAT. This study observed significantly lower density values of L1 and SAT measured in the VNCa, VNCn and VNCv reconstructions compared to the TNC images. These findings may be due to the algorithm on which VNC is based. However, these findings indicate that VNC reconstructions have to be used carefully in adipose and calcified structures. Previous work based on DECT-derived image data was able to show the same deviation between the TNC and VNC images of adipose tissue [27]. Keeping this in mind, the evaluation of adipose structures as hepatosteatosis should be carried out carefully. Nevertheless, Niehoff et al. were able to show that PCCT-derived VNC sequences can be used for the assessment of hepatic steatosis [34]. This indicates that the described differences in adipose tissue might be present but clinical use might still be possible. Looking at calcified structures, there is preliminary work available, especially in the field of cardiac imaging. Several studies have evaluated the usability of VNC reconstructions for calcium score evaluation in cardiac imaging. Using PCCT-derived image data, Sharma et al. demonstrated that calcium scores calculated from VNC images were underestimated [35]. Also, in DECT, the calcium scores calculated from VNC reconstructions seemed to be lower than these calculated from the TNC reconstructions [36,37]. This is well in line with our findings, as the current study also showed significantly lower mean values measured in L1 in the VNC reconstructions compared to the TNC reconstructions. However, it has also been reported that VNC images can be used for calcium score calculations [37,38] even if there is a need for optimisation.

Looking at the structures with contrast media uptake, the presented study was able to show an approximation of the mean density values. The significant differences in the mean values between the TNC and early arterial contrast phase reconstructions measured in the DA and spleen parenchyma could be brought to a non-significant level by creating VNC sequences. Likewise, the significant differences between the mean values of the TNC and portal venous contrast phase reconstructions measured in the PV, ICV, liver parenchyma, spleen parenchyma, PM and ESM were brought to a non-significant level by creating VNC reconstructions. These findings illustrate that VNC reconstructions may allow for the subtraction of the given contrast media and allow for an evaluation of parenchymal organs, muscles and vessels. In line with these results, other studies were able to show that VNC reconstructions can be used to evaluate abdominal structures equally as well as TNC, e.g., for the classification of adrenal masses [27], evaluation of liver tissue [25,39], classification of pancreatic lesions [11] or classification of renal masses [40,41]. Furthermore, it was shown that the VNC reconstructions derived from venous contrast phase might be more accurate than the VNC reconstructions derived from the arterial contrast phase [42,43]. These could neither be refuted nor confirmed by the current study. However, no significant differences between the VNCn, VNCa and VNCv reconstructions could be demonstrated.

The present study now allows for the basic statement that both VNCa and VNCv reconstructions represent an approach to measuring the density values that is similar to TNC reconstructions and equalise the significant differences between them. This supports the external findings listed above, describing the clinical benefits and potential applications of VNC reconstructions in the assessment of parenchymal organs. Nevertheless, according to the demonstrated deviations in the SAT and L1 and the previously described limitations [12,31,32,33], VNC reconstructions should be used carefully. Additionally, the results of the current study show that significant differences in muscle tissue between the TNC and contrast-enhanced scans in the portal venous phase can be diminished by creating VNC reconstructions. This finding suggests that VNCv is a promising addition to the evaluation of muscular tissue in PCCT. In addition, the use of this finding, for example, for the evaluation of muscular haemorrhages, could be investigated in subsequent studies. On the other hand, our results illustrate that VNC cannot completely substitute TNC, which is highlighted by the findings for the SAT and L1. However, there were no significant differences between VNCn, VNCa and VNCv observed.

The current study was limited by its rather small group of enrolled patients, which is caused by the fact that PCCT is relatively new and so far, only a few patients have been examined with this specific protocol. Nevertheless, our study provides a proof of concept and supports existing data that show that VNC can be used carefully in specific clinical settings. However, subsequent studies should enrol a bigger, more heterogenous number of patients. For further studies, it would also be interesting to compare the PureCalcium VNC reconstructions in addition to the conventional VNC reconstructions [44]. Additionally, following studies with a larger patient cohorts can also address the described results in the context of clinical issues such as bleeding examination, the evaluation of vasosclerosis or the detection of kidney stones.

## Figures and Tables

**Table 1:** Patient cohort overview.

*n*	17
Age (mean (SD))	75.65 (9)
Reason for examination	
Staging	9
Vessel examination	7
Bleeding evaluation	1
Sex (%)	
Male	11 (65)
Female	6 (35)

**Table 2:** Measured density mean values with significance levels after comparison to TNC. The density values are given in Hounsfield units. Mean values of native-derived virtual non-contrast (VNCn), early arterial (ea), ea-derived VNC (VNCa), portal venous (pv) and pv-derived VNC (VNCv) were compared to true non-contrast (TNC) by Kruskal–Wallis test. *: *p* < 0.05 (in comparison to mean TNC (Kruskal–Wallis).

	TNC	VNCn	ea	VNCa	pv	VNCv
*n*	17	17	17	17	17	17
Portal vein (mean (SD))	46.71 (19.42)	39.06 (9.42)	76.24 (31.98)	39.12 (7.83)	216.35 * (35.87)	35.00 (7.94)
Descending aorta (mean (SD))	47.94 (18.69)	40.18 (8.15)	411.00 * (126.14)	34.76 (7.45)	195.82 (35.77)	32.18 (7.99)
IVC (mean (SD))	44.76 (18.30)	38.59 (8.03)	59.75 (23.87)	38.82 (8.44)	145.47 * (40.08)	35.06 (8.68)
Liver parenchyma (mean (SD))	62.12 (8.25)	62.65 (10.03)	65.94 (9.74)	61.76 (7.85)	123.35 * (22.76)	62.06 (10.63)
Spleen parenchyma (mean (SD))	54.12 (9.92)	50.47 (7.04)	93.65 * (21.75)	49.59 (8.37)	140.12 * (20.72)	47.35 (9.12)
Erector spinae muscle (mean (SD))	42.71 (11.78)	47.47 (11.90)	43.94 (9.88)	45.24 (9.78)	52.65 * (14.14)	42.82 (11.16)
Psoas muscle (mean (SD))	49.47 (7.80)	48.65 (9.90)	52.00 (7.78)	45.62 (10.37)	60.82 * (8.23)	46.41 (8.35)
Subcutaneous adipose tissue (mean (SD))	−100.82 (10.97)	−76.76 * (10.25)	−111.24 (15.60)	−76.59 * (11.93)	−111.18 (16.98)	−78.94 * (11.29)
Vertebral body (L1), (mean (SD))	83.24 (12.30)	48.94 * (20.14)	104.65 (26.58)	49.47 * (17.97)	121.88 (35.73)	44.41 * (20.61)
Air (mean (SD))	−998.65 (2.91)	−998.88 (2.26)	−998.00 (3.50)	−998.82 (3.40)	−997.47 (4.49)	−999.53 (2.35)

**Table 3:** Listing of *p*-values in comparison to true non-contrast. Abbreviations: native-derived virtual non-contrast (VNCn), early arterial (ea), ea-derived VNC (VNCa), portal venous (pv) and pv-derived VNC (VNCv). a: multiple comparisons were not carried out because the overall test did not show any significant differences between samples. ^b^: after Bonferroni correction.

		Portal Vein					Descending Aorta					IVC				
		VNCn	ea	VNCa	pv	VNCv	VNCn	ea	VNCa	pv	VNCv	VNCn	ea	VNCa	pv	VNCv
**Kruskal–Wallis Test**	*p*	0.456	0.005	0.481	<0.001	0.109	0.485	<0.001	0.038	0.004	0.009	0.580	0.030	0.673	<0.001	0.152
	*p* ^b^	1	0.072	1	<0.001	1	1	<0.001	0.565	0.055	0.134	1	0.455	1	<0.001	1
		**Liver parenchyma**					**Spleen parenchyma**									
		VNCn	ea	VNCa	pv	VNCv	VNCn	ea	VNCa	pv	VNCv					
**Kruskal–Wallis Test**	*p*	0.600	0.342	0.882	<0.001	0.572	0.737	<0.001	0.499	<0.001	0.277					
	*p* ^b^	1	1	1	<0.001	1	1	0.003	1	<0.001	1					
		**Erector spinae muscle**					**Psoas muscle**									
		VNCn	ea	VNCa	pv	VNCv	VNCn	ea	VNCa	pv	VNCv					
**Kruskal–Wallis Test**	*p*	0.137	0.826	0.590	0.003	0.981	0.841	0.345	0.378	<0.001	0.306					
	*p* ^b^	1	1	1	0.040	1	1	1	1	0.004	1					
		**Subcutaneous adipose tissue**					**Vertebral body (L1)**					**Air**				
		VNCn	ea	VNCa	pv	VNCv	VNCn	ea	VNCa	pv	VNCv	VNCn	ea	VNCa	pv	VNCv
**Kruskal–Wallis Test**	*p*	<0.001	0.245	<0.001	0.263	<0.001	<0.001	0.244	<0.001	0.042	<0.001	a	a	a	a	a
	*p* ^b^	<0.001	1	0.002	1	0.006	0.006	1	0.006	0.623	<0.001	a	a	a	a	a

**Table 4:** Mean values and standard deviation (SD) of measured standard deviation. Abbreviations: native-derived virtual non-contrast (VNCn), early arterial (ea), ea-derived VNC (VNCa), portal venous (pv), pv-derived VNC (VNCv) and true non-contrast (TNC).

	TNC	VNCn	ea	VNCa	pv	VNCv
*n*	17	17	17	17	17	17
Portal vein (mean (SD))	19.29 (7.90)	15.65 (4.92)	27.18 (8.46)	15.06 (2.97)	28.76 (10.93)	14.82 (2.88)
Descending aorta (mean (SD))	15.82 (2.48)	11.94 (2.19)	24.35 (4.69)	15.00 (3.14)	22.76 (3.78)	14.12 (2.83)
IVC (mean (SD))	19.53 (9.09)	15.65 (7.37)	24.38 (5.67)	15.18 (3.78)	24.88 (5.02)	13.65 (3.20)
Liver parenchyma (mean (SD))	13.65 (2.94)	10.41 (2.18)	17.76 (3.67)	11.94 (2.59)	18.88 (3.53)	11.18 (2.67)
Spleen parenchyma (mean (SD))	13.47 (3.08)	10.65 (2.69)	23.41 (6.13)	13.47 (4.36)	20.29 (4.40)	12.12 (3.26)
Erector spinae muscle (mean (SD))	17.71 (5.21)	13.18 (4.98)	22.35 (6.41)	14.18 (4.07)	21.94 (5.23)	13.94 (3.33)
Psoas muscle (mean (SD))	16.12 (2.71)	13.06 (2.73)	20.88 (3.54)	13.31 (2.63)	22.12 (4.36)	14.06 (3.01)
Subcutaneous adipose tissue (mean (SD))	15.94 (6.68)	12.53 (4.09)	16.76 (5.14)	12.53 (2.92)	16.94 (4.15)	12.35 (2.03)
Vertebral body (L1), (mean (SD))	24.29 (6.31)	15.12 (4.18)	33.29 (12.89)	18.35 (6.31)	31.65 (10.03)	16.24 (4.91)
Air (mean (SD))	8.82 (3.59)	7.12 (2.62)	12.71 (4.54)	8.76 (3.73)	13.12 (5.19)	8.41 (2.96)

## Data Availability

The data presented in this study are available on request from the corresponding author.

## Abbreviations

CT	Computed tomography
DA	Descending aorta
ESM	Erector spinae muscle
HU	Hounsfield units
IVC	Inferior vena cava
L1	First lumbar vertebral body
PCCT	Photon-counting computed tomography
PM	Psoas muscle
PV	Portal vein
ROI	Regions of interest
SAT	Subcutaneous adipose tissue
SD	Standard deviation
TNC	True non-contrast
VNC	Virtual non-contrast
VNCa	Virtual non-contrast (derived from early arterial contrast phase)
VNCn	Virtual non-contrast (derived from native contrast phase)
VNCv	Virtual non-contrast (derived from portal venous contrast phase)

## References

[1] Rajendran K., Petersilka M., Henning A., Shanblatt E.R., Schmidt B., Flohr T.G., Ferrero A., Baffour F., Diehn F.E., Yu L. (2022). First Clinical Photon-Counting Detector CT System: Technical Evaluation. Radiology.

[2] Pannenbecker P., Huflage H., Grunz J.-P., Gruschwitz P., Patzer T.S., Weng A.M., Heidenreich J.F., Bley T.A., Petritsch B. (2023). Photon-counting CT for diagnosis of acute pulmonary embolism: Potential for contrast medium and radiation dose reduction. Eur. Radiol..

[3] Greffier J., Villani N., Defez D., Dabli D., Si-Mohamed S. (2023). Spectral CT imaging: Technical principles of dual-energy CT and multi-energy photon-counting CT. Diagn. Interv. Imaging.

[4] Tharmaseelan H., Rotkopf L.T., Ayx I., Hertel A., Nörenberg D., Schoenberg S.O., Froelich M.F. (2022). Evaluation of radiomics feature stability in abdominal monoenergetic photon counting CT reconstructions. Sci. Rep..

[5] Rajiah P., Parakh A., Kay F., Baruah D., Kambadakone A.R., Leng S. (2020). Update on Multienergy CT: Physics, Principles, and Applications. Radiographics.

[6] Kahn J., Fehrenbach U., Böning G., Feldhaus F., Maurer M., Renz D., Streitparth F. (2019). Spectral CT in patients with acute thoracoabdominal bleeding—A safe technique to improve diagnostic confidence and reduce dose?. Medicine.

[7] Kessner R., Sommer J., Große Hokamp N., Laukamp K.R., Nayate A. (2023). Virtual versus true non-contrast images of the brain from spectral detector CT: Comparison of attenuation values and image quality. Acta Radiol..

[8] Javadi S., Elsherif S., Bhosale P., Jensen C.T., Layman R.R., Jacobsen M.C., Le O., Jia S., Parikh R.J., Tamm E.P. (2020). Quantitative attenuation accuracy of virtual non-enhanced imaging compared to that of true non-enhanced imaging on dual-source dual-energy CT. Abdom. Radiol..

[9] Laukamp K.R., Lennartz S., Ho V., Große Hokamp N., Zopfs D., Gupta A., Graner F.P., Borggrefe J., Gilkeson R., Ramaiya N. (2020). Evaluation of the liver with virtual non-contrast: Single institution study in 149 patients undergoing TAVR planning. Br. J. Radiol..

[10] Bucolo G.M., Ascenti V., Barbera S., Fontana F., Aricò F.M., Piacentino F., Coppola A., Cicero G., Marino M.A., Booz C. (2023). Virtual Non-Contrast Spectral CT in Renal Masses: Is It Time to Discard Conventional Unenhanced Phase?. J. Clin. Med..

[11] Liang H., Du S., Yan G., Zhou Y., Yang T., Zhang Z., Luo C., Liao H., Li Y. (2023). Dual-energy CT of the pancreas: Comparison between virtual non-contrast images and true non-contrast images in the detection of pancreatic lesion. Abdom. Radiol..

[12] Lee M.H., Park H.J., Kim J.N., Kim M.S., Hong S.W., Park J.H., Kang C.H. (2022). Virtual non-contrast images from dual-energy CT angiography of the abdominal aorta and femoral arteries: Comparison with true non-contrast CT images. Br. J. Radiol..

[13] Si-Mohamed S., Dupuis N., Tatard-Leitman V., Rotzinger D., Boccalini S., Dion M., Vlassenbroek A., Coulon P., Yagil Y., Shapira N. (2019). Virtual versus true non-contrast dual-energy CT imaging for the diagnosis of aortic intramural hematoma. Eur. Radiol..

[14] Song I., Yi J.G., Park J.H., Kim S.M., Lee K.S., Chung M.J. (2016). Virtual Non-Contrast CT Using Dual-Energy Spectral CT: Feasibility of Coronary Artery Calcium Scoring. Korean J. Radiol..

[15] Mergen V., Ghouse S., Sartoretti T., Manka R., Euler A., Kasel A.M., Alkadhi H., Eberhard M. (2023). Cardiac Virtual Noncontrast Images for Calcium Quantification with Photon-Counting Detector CT. Radiol. Cardiothorac. Imaging.

[16] Al-Difaie Z., Scheepers M.H., Bouvy N.D., Engelen S., Havekes B., Postma A.A. (2023). Can virtual non-contrast imaging replace true non-contrast imaging in multiphase scanning of the neck region?. Acta Radiol. Open.

[17] Reimer R.P., Zaytoun H., Klein K., Sonnabend K., Lennartz S., Zopfs D., Heidenreich A., Maintz D., Große Hokamp N. (2023). Detection and size measurements of kidney stones on virtual non-contrast reconstructions derived from dual-layer computed tomography in an ex vivo phantom setup. Eur. Radiol..

[18] Schoenbeck D., Pauline Haag N., Elias Michael A., Michael Woeltjen M., Boriesosdick J., Saeed S., Borggrefe J., Robert Kroeger J., Henning Niehoff J. (2023). Dedicated virtual non-contrast images adapted for liver tissue in clinical photon counting CT improve virtual non-contrast imaging in various organs beyond the liver. Eur. J. Radiol..

[19] Niehoff J.H., Woeltjen M.M., Laukamp K.R., Borggrefe J., Kroeger J.R. (2021). Virtual Non-Contrast versus True Non-Contrast Computed Tomography: Initial Experiences with a Photon Counting Scanner Approved for Clinical Use. Diagnostics.

[20] Rickham P.P. (1964). Human Experimentation. Code of Ethics of the World Medical Association. Declaration of Helsinki. Br. Med. J..

[21] R Core Team (2022). R: A Language and Environment for Statistical Computing. R Foundation for Statistical Computing. https://www.r-project.org/.

[22] Reginelli A., Capasso R., Ciccone V., Croce M.R., Di Grezia G., Carbone M., Maggialetti N., Barile A., Fonio P., Scialpi M. (2016). Usefulness of triphasic CT aortic angiography in acute and surveillance: Our experience in the assessment of acute aortic dissection and endoleak. Int. J. Surg..

[23] Cassagnes L., Pérignon R., Amokrane F., Petermann A., Bécaud T., Saint-Lebes B., Chabrot P., Rousseau H., Boyer L. (2016). Aortic stent-grafts: Endoleak surveillance. Diagn. Interv. Imaging.

[24] Brisbane W., Bailey M.R., Sorensen M.D. (2016). An overview of kidney stone imaging techniques. Nat. Rev. Urol..

[25] Zhang P.P., Choi H.H., Ohliger M.A. (2022). Detection of fatty liver using virtual non-contrast dual-energy CT. Abdom. Radiol..

[26] Ma J., Song Z.-Q., Yan F.-H. (2014). Separation of hepatic iron and fat by dual-source dual-energy computed tomography based on material decomposition: An animal study. PLoS ONE.

[27] Winkelmann M.T., Gassenmaier S., Walter S.S., Artzner C., Lades F., Faby S., Nikolaou K., Bongers M.N. (2022). Differentiation of adrenal adenomas from adrenal metastases in single-phased staging dual-energy CT and radiomics. Diagn. Interv. Radiol..

[28] Dodig D., Solocki Matić T., Žuža I., Pavlović I., Miletić D., Markić D. (2021). Side-by-side evaluation of virtual non-contrast and post-contrast images improves detection of clinically significant urolithiasis on single-phase split bolus dual-energy CT urography. Br. J. Radiol..

[29] McCoombe K., Dobeli K., Meikle S., Llewellyn S., Kench P. (2022). Sensitivity of virtual non-contrast dual-energy CT urogram for detection of urinary calculi: A systematic review and meta-analysis. Eur. Radiol..

[30] Winkelmann M.T., Hagen F., Artzner K., Bongers M.N., Artzner C. (2022). Dual-Energy CT for Accurate Discrimination of Intraperitoneal Hematoma and Intestinal Structures. Diagnostics.

[31] Lehti L., Söderberg M., Höglund P., Nyman U., Gottsäter A., Wassélius J. (2018). Reliability of virtual non-contrast computed tomography angiography: Comparing it with the real deal. Acta Radiol. Open.

[32] Yun S.Y., Heo Y.J., Jeong H.W., Baek J.W., Choo H.J., Shin G.W., Kim S.T., Jeong Y.G., Lee J.Y., Jung H.S. (2019). Dual-energy CT angiography-derived virtual non-contrast images for follow-up of patients with surgically clipped aneurysms: A retrospective study. Neuroradiology.

[33] Durieux P., Gevenois P.A., van Muylem A., Howarth N., Keyzer C. (2018). Abdominal Attenuation Values on Virtual and True Unenhanced Images Obtained with Third-Generation Dual-Source Dual-Energy CT. AJR Am. J. Roentgenol..

[34] Niehoff J.H., Woeltjen M.M., Saeed S., Michael A.E., Boriesosdick J., Borggrefe J., Kroeger J.R. (2022). Assessment of hepatic steatosis based on virtual non-contrast computed tomography: Initial experiences with a photon counting scanner approved for clinical use. Eur. J. Radiol..

[35] Sharma S.P., van der Bie J., van Straten M., Hirsch A., Bos D., Dijkshoorn M.L., Booij R., Budde R.P.J. (2023). Coronary calcium scoring on virtual non-contrast and virtual non-iodine reconstructions compared to true non-contrast images using photon-counting computed tomography. Eur. Radiol..

[36] Yang P., Zhao R., Deng W., An S., Li Y., Sheng M., Chen X., Qian Y., Yu Y., Mu D. (2023). Feasibility and accuracy of coronary artery calcium score on virtual non-contrast images derived from a dual-layer spectral detector CT: A retrospective multicenter study. Front. Cardiovasc. Med..

[37] Nadjiri J., Kaissis G., Meurer F., Weis F., Laugwitz K.-L., Straeter A.S., Muenzel D., Noël P.B., Rummeny E.J., Rasper M. (2018). Accuracy of Calcium Scoring calculated from contrast-enhanced Coronary Computed Tomography Angiography using a dual-layer spectral CT: A comparison of Calcium Scoring from real and virtual non-contrast data. PLoS ONE.

[38] Gassert F.G., Schacky C.E., Müller-Leisse C., Gassert F.T., Pahn G., Laugwitz K.-L., Makowski M.R., Nadjiri J. (2021). Calcium scoring using virtual non-contrast images from a dual-layer spectral detector CT: Comparison to true non-contrast data and evaluation of proportionality factor in a large patient collective. Eur. Radiol..

[39] Choi M.H., Lee Y.J., Choi Y.J., Pak S. (2021). Dual-energy CT of the liver: True noncontrast vs. virtual noncontrast images derived from multiple phases for the diagnosis of fatty liver. Eur. J. Radiol..

[40] Park S.Y., Kim C.K., Park B.K. (2014). Dual-energy CT in assessing therapeutic response to radiofrequency ablation of renal cell carcinomas. Eur. J. Radiol..

[41] Lin Y.-M., Chiou Y.-Y., Wu M.-H., Huang S.-S., Shen S.-H. (2018). Attenuation values of renal parenchyma in virtual noncontrast images acquired from multiphase renal dual-energy CT: Comparison with standard noncontrast CT. Eur. J. Radiol..

[42] Lehti L., Söderberg M., Höglund P., Wassélius J. (2019). Comparing Arterial- and Venous-Phase Acquisition for Optimization of Virtual Noncontrast Images from Dual-Energy Computed Tomography Angiography. J. Comput. Assist. Tomogr..

[43] Risch F., Bette S., Sinzinger A., Rippel K., Scheurig-Muenkler C., Kroencke T., Decker J.A. (2023). Multiphase photon counting detector CT data sets—Which combination of contrast phase and virtual non-contrast algorithm is best suited to replace true non-contrast series in the assessment of active bleeding?. Eur. J. Radiol..

[44] Wildberger J.E., Alkadhi H. (2023). New Horizons in Vascular Imaging with Photon-Counting Detector CT. Investig. Radiol..

